# Slowly Adapting Sensory Units Have More Receptors in Large Airways than in Small Airways in Rabbits

**DOI:** 10.3389/fphys.2016.00588

**Published:** 2016-12-09

**Authors:** Jun Liu, Nana Song, Juan Guardiola, Jesse Roman, Jerry Yu

**Affiliations:** Pulmonary Division, Department of Medicine, University of Louisville; and Robley Rex VA Medical CenterLouisville, KY, USA

**Keywords:** vagus nerve, sensory unit, sensory receptor cells, sensory receptor, lung afferents, airway receptor, airway sensors

## Abstract

Sensory units of pulmonary slowly adapting receptors (SARs) are more active in large airways than in small airways. However, there is no explanation for this phenomenon. Although sensory structures in large airways resemble those in small airways, they are bigger and more complex. Possibly, a larger receptor provides greater surface area for depolarization, and thus has a lower activating threshold and/or a higher sensitivity to stretch, leading to more nerve electrical activities. Recently, a single sensory unit has been reported to contain multiple receptors. Therefore, sensory units in large airways may contain more SARs, which may contribute to high activities. To test this hypothesis, we used a double staining technique to identify sensory receptor sizes. We labeled the sensory structure with Na^+^/K^+^-ATPase antibodies and the myelin sheath with myelin basic protein (MBP) antibodies. A SAR can be defined as the end formation beyond MBP labeling. Thus, we are able to compare sizes of sensory structures and SARs in large (trachea and bronchi) vs. small (bronchioles <500 μm in diameter) airways in the rabbit. We found that even though the sensory structure was bigger in large airways than in small airways (3340 ± 223 vs. 1168 ± 103 μm^2^; *P* < 0.0001), there was no difference in receptor sizes (349 ± 14 vs. 326 ± 16 μm^2^; > 0.05). However, the sensory structure contains more SARs in large airways than in small airways (9.6 ± 0.6 vs. 3.6 ± 0.3; *P* < 0.0001). Thus, our data support the hypothesis that greater numbers of SARs in sensory units of large airways may contribute to higher activities.

## Introduction

Information from airway sensory receptors or sensors to the brain is mainly carried via the vagus nerve and yields responses under physiological and pathophysiological conditions. However, little is known about the receptor structure (von Düring et al., 1974; Krauhs, 1984; Baluk and Gabella, 1991; Yamamoto et al., 1995; Wang and Yu, 2002), and even less about receptor structure-function relationships. Such information is required to fully understand the function of these receptors. With advances in immunohistochemistry, neural tracing, and microscopic techniques, the airway sensory structure can be examined in detail and evaluated objectively. An excellent marker (Na^+^/K^+^-ATPase) for airway sensors has been identified (Wang and Yu, 2002). Using this biomarker, structures of slowly adapting receptors (SARs) in the airways have been examined extensively in rats (Adriaensen et al., 2006; Matsumoto et al., 2006), guinea pigs (Mazzone et al., 2009), and rabbits (Wang and Yu, 2004)^1^. The discovery of multiple receptive fields in a single unit (Yu and Zhang, 2004), along with multiple sensory structures connected to a single axon (Yu et al., 2003), has prompted a theory stating that mechanosensory units are functional units that contain multiple receptors (Yu, 2005). In the airways, SARs can be divided into two types, low-threshold (with discharge activity during expiration) and high-threshold (silent during expiration; Paintal, 1973; Coleridge and Coleridge, 1986). More low-threshold SARs were located in the central airways, whereas more high-threshold SARs were located in the peripheral airways (Ravi, 1986) (in cats). Since SARs are more active in large airways than in small airways, it is possible that larger SARs give a lower activating threshold or a higher sensitivity to stretch (Ravi, 1986; Yu et al., 1991). Using the Na^+^/K^+^-ATPase antibody, we found that sensory structures were larger in large airways than in small airways, leading us to conclude that higher activities of SARs in the large airways may result from larger sensory structures (Liu et al., 2012). However, it is still unanswered if the larger sensory structure is caused by a greater number of receptors or by bigger size of receptors, or by both. Using double labeling with antibodies against Na^+^/K^+^-ATPase and myelin basic protein (MBP), we are able to examine receptor size. Therefore, we set out to characterize sensory structures in the large vs. small airways by comparing receptor sizes.

## Methods

Current studies conformed to the Guide for the Care and Use of Laboratory Animals published by the United States National Institutes of Health (NIH Publication No. 85-53). The Institutional Animal Care and Use Committee at University of Louisville and the Robley Rex VA Medical Center approved the use of animals and the study protocol.

Ten young adult male New Zealand White rabbits (1.5–2.0 kg) were sacrificed by anesthesia with ketamine/xylazine (40/10 mg/kg) IM, which was followed by an overdose of saturated KCl IV to arrest the heart. Airways were obtained immediately after euthanasia and fixed overnight in a 0.1 M Phosphate Buffered Saline (PBS) containing 4% paraformaldehyde (at pH 7.4). About 1–2 segments from large airways (tracheal smooth muscles) and 5–10 segments from small airways (bronchioles <500 μm in diameter) were used for staining, and images with high quality of fluorescent structures were used for analysis. Airways were isolated and dissected in PBS for double-label immune-histochemical procedures. Whole mount tissue preparations were washed in PBS three times for 10 min (total 30 min) and then washed in PBS containing 0.4% Triton X-100 hourly for 6 h, followed by blocking for 2 h in PBS containing 5% normal serum and 3% bovine serum albumin. Preparations were then incubated overnight with mouse monoclonal antibody (Anti-Na^+^/K^+^-ATPase, α3 subunit; Enzo Life Sciences, Inc. NY; diluted to 1:200) and chicken polyclonal anti-MBP (AVES Labs, Inc. OR, USA; diluted to 1:100) at 4°C. The preparations were then washed with PBS and incubated with cy3-labeled donkey anti-mouse immunoglobulin G (Jackson Immuno Research; diluted at 1:100) and Alexa Fluor® 488 goat anti-chicken IgG (Invitrogen corporation, CA, USA; diluted to 1:500) for 60–120 min at room temperature. After a final rinse with PBS, tissues were mounted onto a glass slide with Fluoromount-G (Southern Biotechnology Associates, Inc. AL, USA) for fluorescent microscopy. The images were recorded for off-line processing. The preparations under investigation had clean background, with the receptor structures clearly labeled.

**Figure 1:**
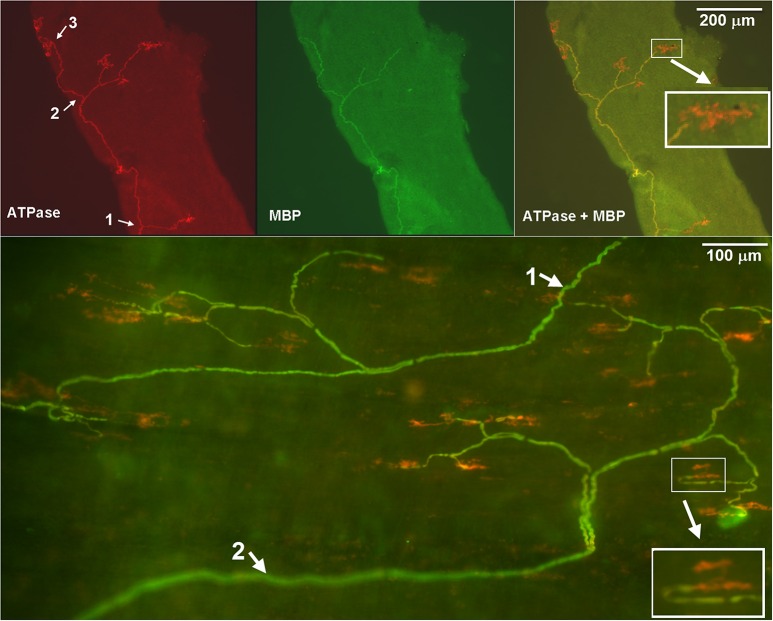
**A double staining approach to illustrate SAR sensory structures identified in rabbit airways**. Na^+^/K^+^-ATPase stains all structures in the sensory unit (red), whereas myelin basic protein (MBP) stains the myelin sheath (green) and shows yellow (co-staining) in the composite figure (top-right and bottom parts). Clearly, the axon demyelinated before it reaches the end formation. Thus, the receptor can be identified (pure red portions without co-stain with MBP). Top (small airway, 300 μm in diameter): The parent axon of the sensory structure is running from the bottom up. It gives off three branches, indicated by white arrows 1, 2, and 3 in the top-left panel. Its first branch is at the bottom of the figure, the second one in the middle part, and the third one at the upper part. Six receptors can be identified in this microscopic view (1 in the first branch, 3 in the second and 2 in the third). They are showing red on double stain. Bottom (trachea): two parent axons (one starts at up-right and one at low left, indicated by white arrows 1 and 2) can be identified. The up-right sensory structure (1) has 9 receptors and the low-left one (2) has 13 receptors. Insets are enlarged to illustrate the sensory receptors.

Before proceeding further, we would like to define some terms used in this report. A sensory receptor (or sensor) is an encoder, which is the basic device that can generate action potentials (Yu, 2005). A sensory unit consists of many receptors. It is a functional unit that transmits action potentials to the central nervous system. A sensory structure, which is a part of the sensory unit, usually contains several receptors connected by a parent axon observed under a microscope. A sensory unit may have more than one such a structure (Figure 1).

For quantitative analysis, sizes of sensory structures and receptors were identified under a fluorescent microscope (Olympus SZ61). Images were taken and analyzed with the software (Image-Pro Plus), which automatically detected the area of interest by its color and color intensity. If necessary, the area could be adjusted manually. Sizes were measured by their projection areas. We used this 2 dimentional measurement to assess the receptor and structure sizes. For example, the sensory structure size is the total projection areas in red plus yellow in the composite figures (Figure 1, top right for a small airway structure; bottom for two large airway structures). Receptor size is the red part in the composite figure. That is the end formation extended beyond myelinated sheath. The number of such end formations was also counted for each receptor structure. Group data are expressed as mean ± SE. Group comparisons were made by Independent-Samples *t*-test with SPSS software. A *P* < 0.05 was considered to be statistically significant.

## Results

**Figure 2:**
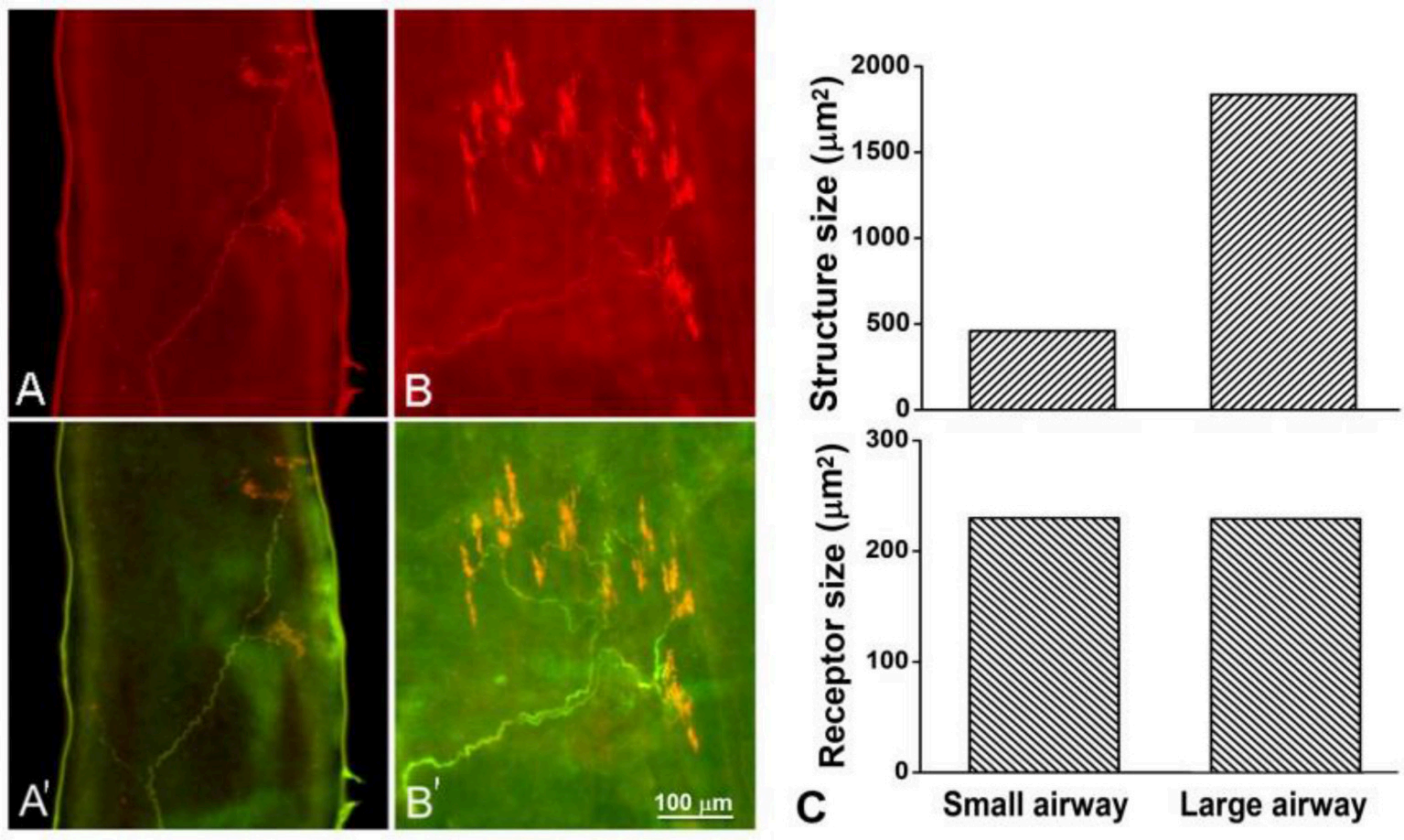
**Comparison of airway receptors in small (A,A′)** and large **(B,B′)** airways. The airway sensory structure was stained with Na^+^-K^+^-ATPase antibody (red); myelin sheath was stained with MBP antibody (green). The structure is bigger in **(B)** than in **(A)**. However, the average sizes of receptors (end formation after nerve demyelination) are the same in small (229.9 μm^2^) and large (229.5 μm^2^) airways **(C)**. There are two receptors in **(A**′**)** and eight receptors in **(B**′**)**.

**Figure 3:**
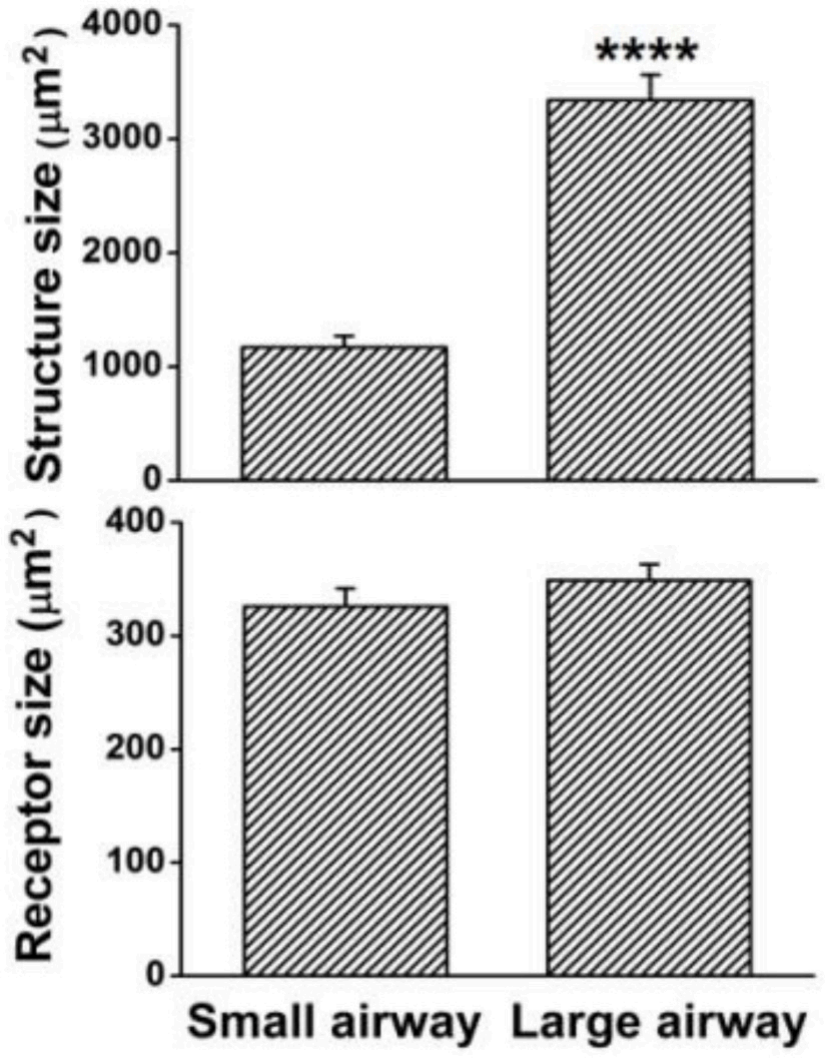
**Group dada for sizes of sensory structures and receptors**. The sensory structure is bigger in large airways (*n* = 16) than in small airways (*n* = 36) (^****^ denotes *P* < 0.0001). However, the receptor sizes were the same in large (*n* = 153) and small (*n* = 129) airways (*P* > 0.05).

**Figure 4:**
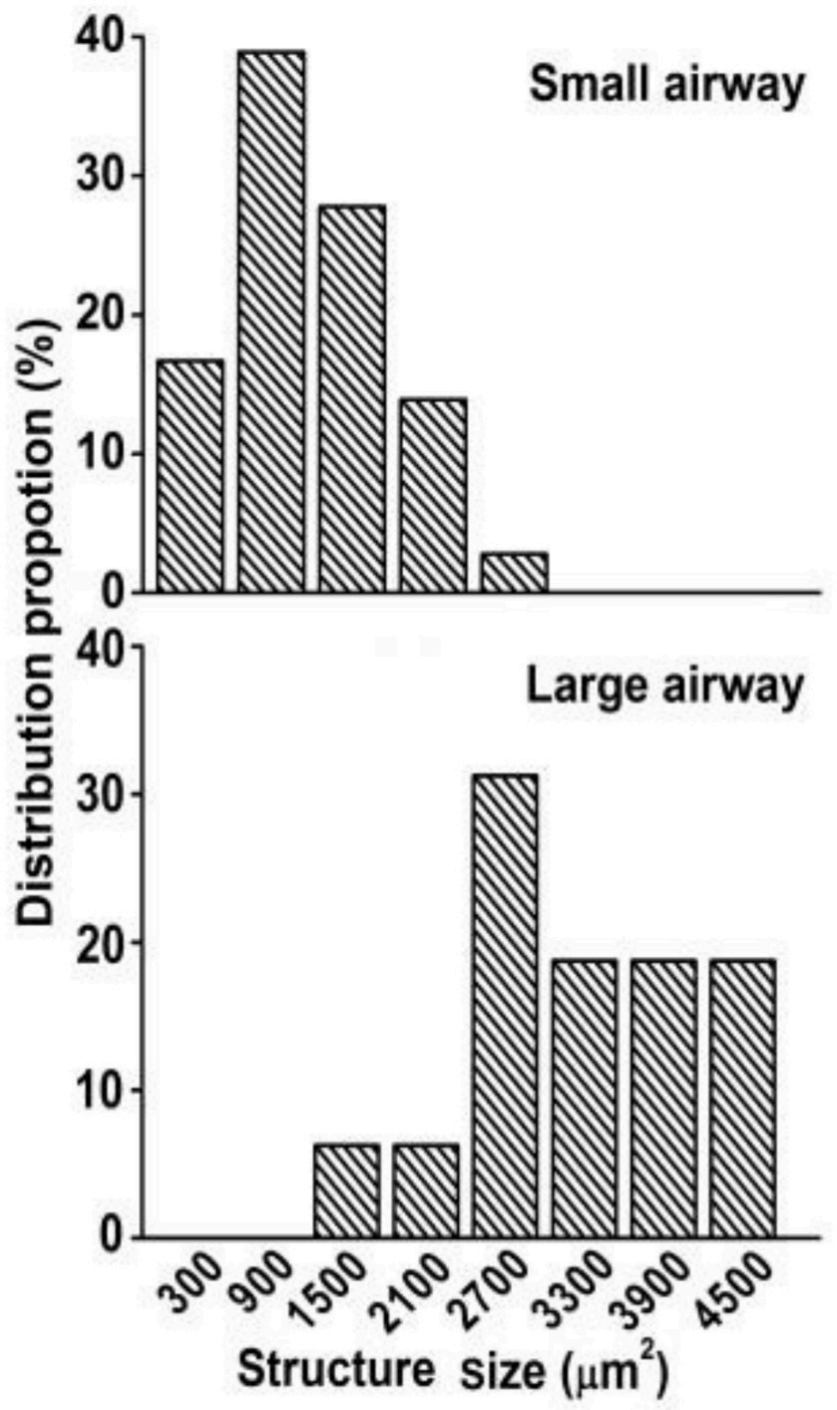
**Size distribution of airway sensory structures (projection area)**. Sizes of 900 and 1500 represent areas of 600–1199 and 1200–1799 μm^2^, respectively. Distribution peaks are 900 and 2700 μm^2^ in small and large airways, respectively. The structure was significantly bigger in large airways (3340 ± 223 μm^2^, *n* = 16) than in small airways (1168 ± 103 μm^2^, *n* = 36; *P* < 0.0001).

**Figure 5:**
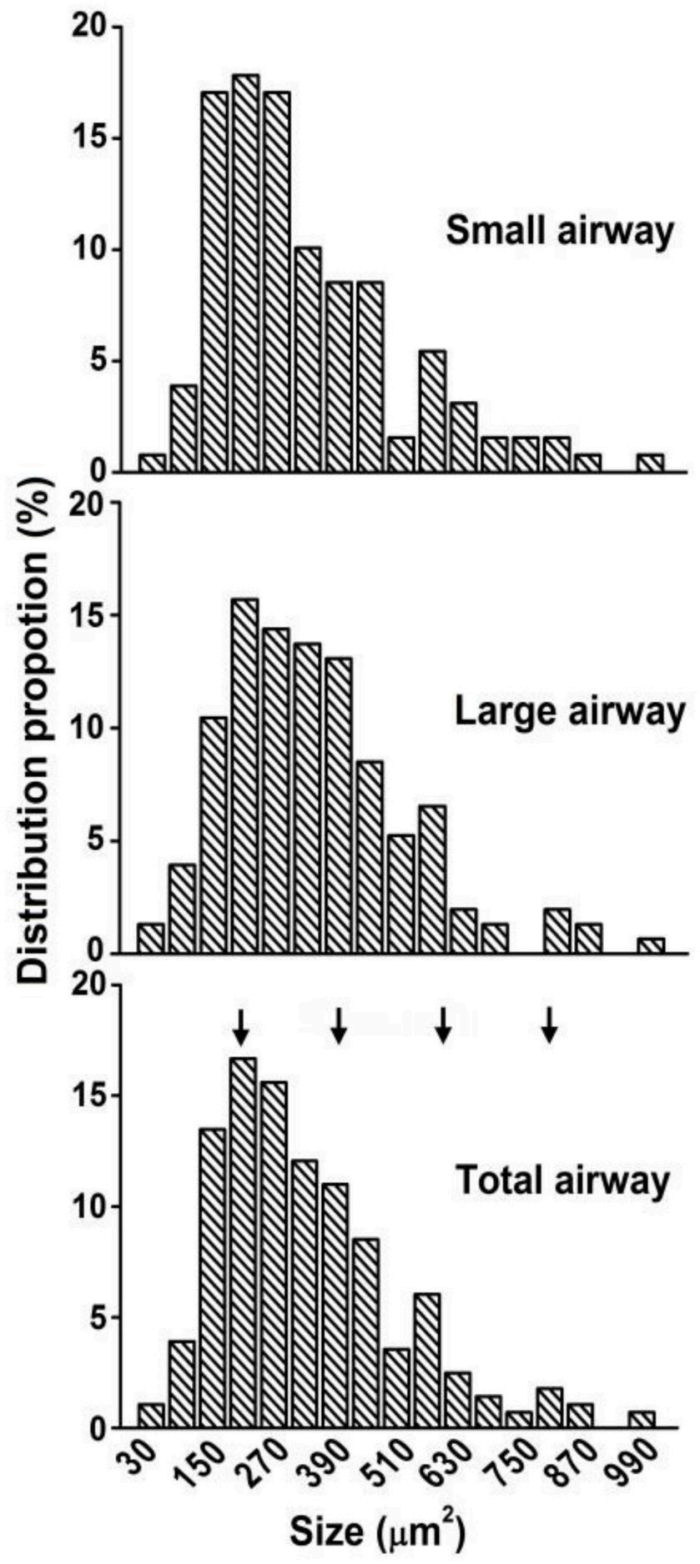
**Distribution of airway receptor size (projection area)**. Sizes of 150 and 270 represent areas of 120–179 and 240–299 μm^2^, respectively. The distribution patterns of receptors in small (*n* = 129) and large (*n* = 153) airways are similar, so that the data are pooled together to show the distribution pattern of total receptors (*n* = 282). The distribution shows 4 peaks at about 200, 400, 600, and 800 μm^2^ (Please see footnote^2^ for details).

**Figure 6:**
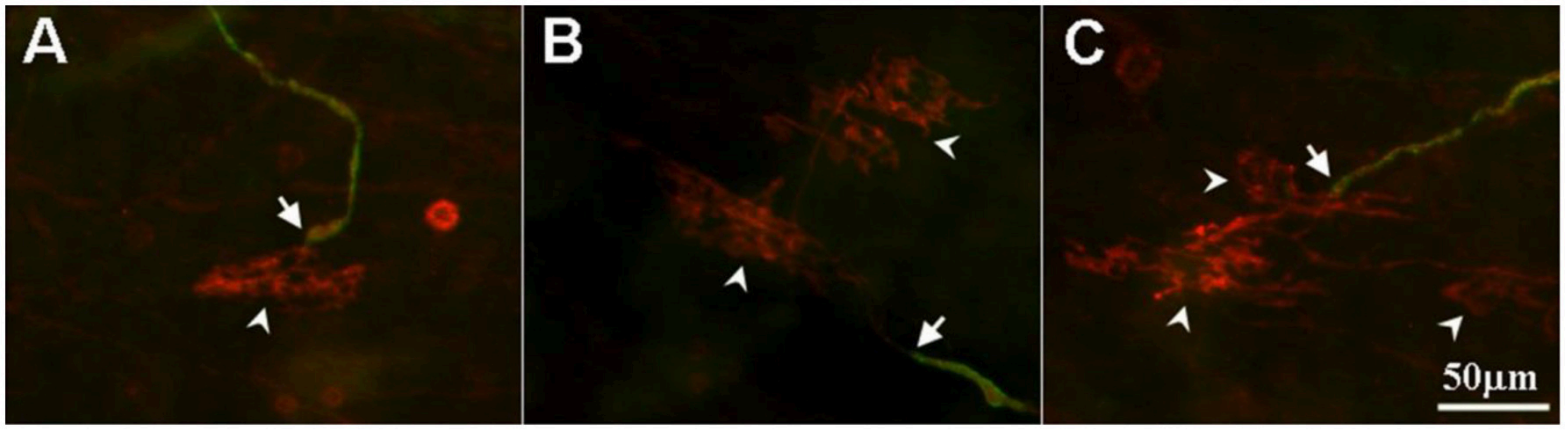
**A receptor (end formation after nerve demyelination) can be singlet (A)**, doublet **(B)** or triplet **(C)**. Airway receptor was stained with Na^+^-K^+^-ATPase antibody (red); myelin sheath was stained with MBP antibody (green). Receptor sizes are: 257 μm^2^ in A, 505 (238 and 267) μm^2^ in B, and 719 (278, 222, and 219) μm^2^ in **(C)**. Arrows show the point of nerve demyelination; arrow heads show a single end formation.

Using the double staining technique, we observed that airway sensory structures resembled those reported previously (Baluk and Gabella, 1991; Wang and Yu, 2004). An axon gave off branches to individual sensory endings that form knob-like or leaf-like extensions (i.e., receptors or sensors; Figures 1, 2). Furthermore, we were able to identify individual sensory receptors, which are the structural parts that extend beyond the point of myelination. Therefore, we were able to compare the sizes of sensory structures and receptors. In these whole mount preparations, we found that sensory structures were bigger in large airways when compared to small airways (Figures 2A–C). The bigger structure resulted from more sensory receptors rather than from bigger receptors (Figures 2A′,B′,C). Figure 3 illustrates the group data. The sensory structures examined contained more SARs in the large airways (9.6 ± 0.6, *n* = 16) than the small ones (3.6 ± 0.3, *n* = 36; *P* < 0.0001). Although there was an overlap in size, on average the sensory structure was bigger in large airways (3340 ± 223 μm^2^, *n* = 16) than in small airways (1168 ± 103 μm^2^, *n* = 36; *P* < 0.0001) (Figures 3, 4). Receptor sizes varied significantly. However, their distribution patterns were the same (Figure 5) and there was no difference between their averaged sizes in large and small airways (Figure 3). Averaged receptor sizes were 349 ± 14 μm^2^ (*n* = 153) in large airways and 326 ± 16 μm^2^ (*n* = 129, *P* > 0.05) in small airways. It is interesting to note that four peaks could be identified in the distribution diagram (Figure 5)^2^, indicating potential differences. Indeed, we found that a sensor may contain more than one subunit. It could be a singlet, doublet or triplet (Figure 6).

**Figure 7:**
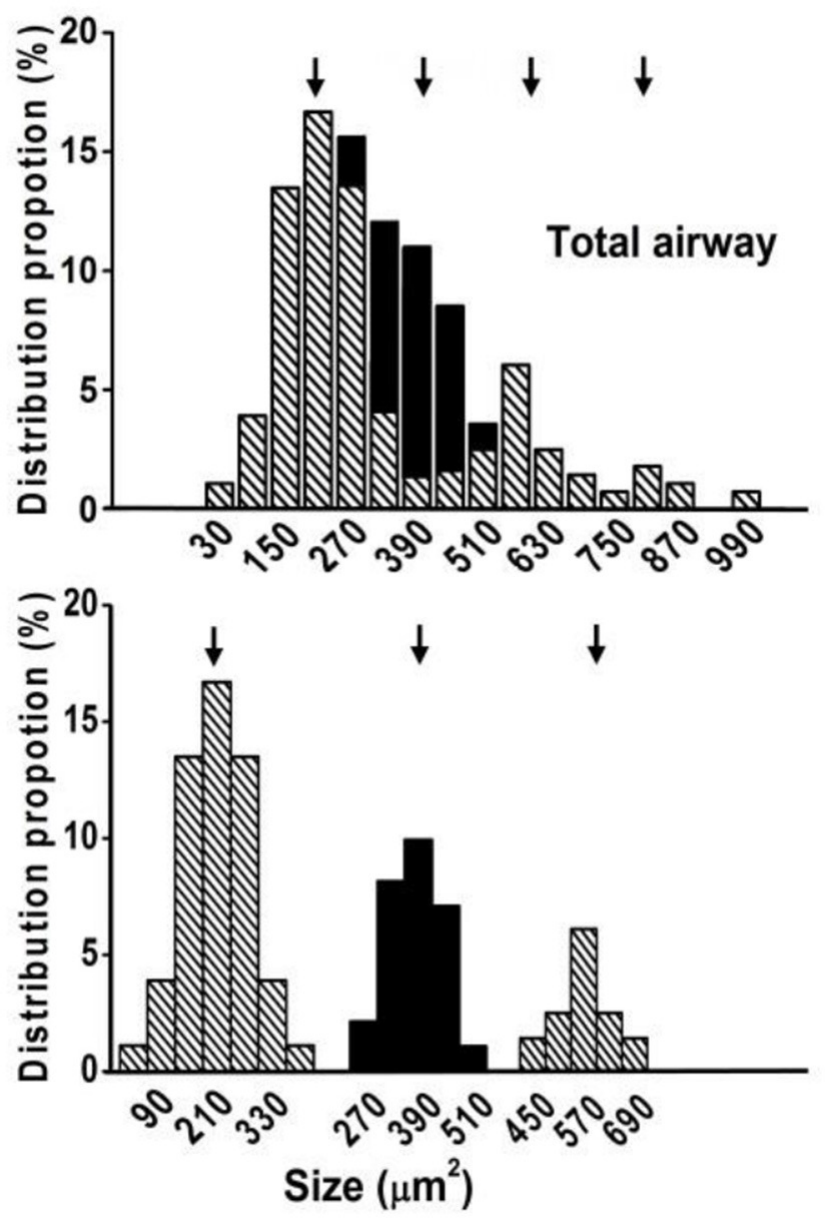
**Illustration of graphical identification of four peaks**. The upper panel is adapted from Figure 5. The lower panel shows the first three peaks. In the upper panel, subtracting the black portions (columns 270, 330, and 390 μm^2^ according to columns 150, 90 and 30 μm^2^, and 450 and 510 μm^2^ according to 690 and 630 μm^2^) results in 2 normal distribution peaks roughly at 200 and 600 μm^2^ (the first and third peaks in the lower panel). The black portion in columns 270, 330, and 390 is obtained by subtracting columns 150, 90 and 30 from 270, 330 and 390, respectively. The five black columns may represent another group of receptors with a peak at 400 μm^2^ (the second peak in the lower panel).

## Discussion

Respiratory centers are under constant influence of afferent signals from the lungs. Airway sensory receptors provide important information to regulate breathing, especially in cardiopulmonary diseases such as heart failure, acute respiratory distress syndrome (ARDS), chronic obstructive pulmonary disease (COPD), and asthma. However, the receptor structure-function relationship is still limited. Our current studies investigated how structural differences in SAR units in large and small airways may contribute to differences in discharge behavior. We demonstrated that the sizes of SARs were the same in different airway segments, however, there were more SARs in sensory units in larger airways. Thus, a large sensory unit, with more SARs, may contribute to the difference in discharge behavior between large and small airways.

Low-threshold SARs are found more frequent in large airways, whereas high-threshold SARs are more common in small airways (Ravi, 1986). The low-threshold SARs may discharge at higher frequencies (Farber et al., 1983; Yu et al., 1991; Davies et al., 1996). It is generally believed that the difference in location of these sensory units accounts for the differences in behavior (Sant'Ambrogio, 1982). However, there is no discussion on the contribution of sensory morphology. This is due to the limited knowledge available on how these receptors operate, in part because the morphology of the sensory unit has not been fully characterized. It has been reported that sensory structures in large airways, although more complex, resemble those in small airways (Baluk and Gabella, 1991). Consistent with this report, using labeling with Na^+^/K^+^-ATPase, we found that sensory structures were similar in their basic formation. However, although they overlapped in size, they were bigger in large airways (5040 ± 826 μm^2^, *n* = 11) than in small airways (2474 ± 577 μm^2^, *n* = 13, *P* < 0.05) (Liu et al., 2012). Thus, there is a difference in sensory morphology in different airways of different sizes.

Greater size or more number of receptors in a unit may result in a low activating threshold and higher discharge frequency. Action potentials are generated from generator potentials (Yu, 2005), which are, in turn, determined by the local potential on the sensing surface of the receptor. Because of summation, the larger the surface of a receptor, the greater the generator potential and the discharge frequency, and the lower the activating threshold. Alternatively, a greater number of receptors in a sensory unit may cause the same behavior because the discharge frequency of a sensory unit is determined by the pacemaker (i.e., the highest discharging receptor). For example, each receptor may discharge at one of 10 possible frequencies (110, 120, 130 …200 Hz) with equal probability (1/10). If there are two units, one with two receptors and one with eight (taking the two units in the Figure 2 as examples), the latter is several folds more likely to contain a higher frequency receptor producing more impulses. By the same token, the latter may discharge at a lower threshold. In current studies, we found that receptor sizes were the same (Figures 2, 3, 5) in large and small airways, however, the sensory structures contained more receptors and therefore, were bigger in larger airways (Figures 2–4).

SARs are connected with myelinated fibers. The myelin sheath is a multi-layered membrane that functions as an insulator to increase the axonal conduction velocity. Myelin basic protein (MBP) can be used to identify myelinated fibers and localize the potential generating site (PGS), where the sensory ending demyelinates (Yu, 2005). In motor neurons, the action potential is generated in the first node where the myelin sheath begins. Similarly, in sensory neurons, the PGS is located at the first node where abundant voltage-dependent sodium channels exist (Yu, 2005). Thus, using MBP as a marker we were able to identify individual receptors in a sensory unit, which are the sensory terminal knobs (Figures 1, 2). The receptor is a basic sensory device that can independently generate action potentials (Yu, 2005). The sensory structures in Figures 1, 2 are only parts of an SAR unit. Unit activity recorded may come from many such structures. In other words, a main sensory axon receives information from several receptive fields (Yu and Zhang, 2004). Conversely, more than one sensory unit may be present in a sensory field (see bottom part of the Figure 1, where two sensory units may co-exist in the field). It needs to be mentioned that with current staining technique RARs may also be included in the studies. However, this should not affect our data interpretation (please see Footnote^1^ for details).

Our current results also show that a sensory receptor may contain subunits. In Figure 5, there are four peaks in the distribution of receptor sizes, indicating that different types of receptors may exist. This is supported by our observation that receptors may contain singlet, doublet, or triplet subunits (Figure 6), which explains why the peaks were roughly equally spaced about 200 μm^2^ apart. For example, the 200 and 600 μm^2^ receptors may be singlets and triplets, respectively. While we do not know the functional difference between singlet and triplet, it is possible that triplets are easier to discharge with a low activating threshold and/or with a high frequency. So far, we emphasized receptor structure in relation to its behavior. However, activation of a receptor is very complicated. There are multiple factors that may affect this process. For example, receptor location and its immediate environment are important since receptors are activated through mechanical coupling with their surroundings (Sant'Ambrogio, 1982; Coleridge and Coleridge, 1986). Nevertheless, our current studies demonstrate that structure may also contribute to discharge behavior of the sensory unit. Future studies will examine receptor morphology and its environment along with electrical activities (threshold, maximal discharge frequency, and slope of activity related to airway pressure) to delineate the underlying mechanism of sensory behavior.

In summary, the current studies show that each airway receptor may contain a different number of subunits, thus varying greatly in size. However, receptor structure, size distribution and averaged size are very similar in large and small airways. In the larger airways, sensory structures contain more receptors, therefore they are larger and more complex. These differences may contribute to their variances in the discharge behavior. Our data support the theory that significant information is integrated at intra- and inter-receptor levels, resulting in final output from a sensory unit to the central nervous system. Thus, airway sensory units function not only as transducers, but also as processors.

## Author contributions

JL conducting experiment, data analysis, writing. NS conducting experiment, data analysis, writing. JG discussion and editing. JR discussion and editing. JY participated in all aspects of the research processes.

## Funding

This study was supported in part by US Department of Veterans Affairs Merit Review Award PULM-029-10S.

### Conflict of interest statement

The authors declare that the research was conducted in the absence of any commercial or financial relationships that could be construed as a potential conflict of interest.

## Footnotes

^1^Both reviewers questioned that whether rapidly adapting receptors (RARs) were also stained. We believe that our current staining technique will label RARs also. Thus, our data may include some RARs. However, this cannot affect our conclusion. First of all, the structure patterns studied are the same as those of SARs, which have been physiologically verified. We only include RARs if they have very similar structures to SARs. Secondly, SARs significantly outnumber RARs at the ratio from 4:1 to 10:1 (Sant'Ambrogio, 1982). The chance of inclusion of RARs is about 10–25%. Thirdly, the distribution pattern of SARs and RARs is quite different. SARs are mainly located in large and small airways, whereas RARs are mainly located in the medium sized airways. For example, 45 and 18% of SARs, and 14 and 5% of RARs are located in the trachea and small airways (<1 mm in diameter), respectively (Sant'Ambrogio, 1982). Thus, the chance of RAR inclusion is reduced by a factor of 3 to give 3–8%. Furthermore, RARs are also believed to be distributed around the circumference of the trachea, while SARs are located in tracheal smooth muscle (Sant'Ambrogio, 1982), the source of our whole mount tissues. Thus, the chance of our data contaminated by RARs is negligible.

^2^Both reviewers asked how four peaks were identified. Four peaks in distribution of receptor sizes is our interpretation. It peaks at about 200 μm^2^. The data are skewed with a clear blip at sizes about 600 μm^2^. This could be an error caused by unexpected reason. Alternatively, many biological events take normal distribution. A normal distribution pattern for 2 groups of receptors with 200 and 600 μm^2^ would be similar to the first and third peaks in the Figure 7, which were obtained after the black portion was subtracted. This black portion can be another group of receptors with sizes around 400 μm^2^ (Figure 7). We can further image a fourth peak at 800 μm^2^. We observed singlets, doublets, and triplets, which could explain the receptor distribution pattern. Please also note that the first peak is the highest and distribution proportions progressively decrease with the peaks, indicating that singlets are most abundant and quadruplets least abundant. We believe this is an interesting observation with a reasonable explanation. However, this needs confirmation by further studies.
